# Effects of vitamin D supplementation on inflammatory markers in heart failure: a systematic review and meta-analysis of randomized controlled trials

**DOI:** 10.1038/s41598-018-19708-0

**Published:** 2018-01-18

**Authors:** Alexander J. Rodriguez, Aya Mousa, Peter R. Ebeling, David Scott, Barbora de Courten

**Affiliations:** 1 Bone and Muscle Health Research Group, Department of Medicine, School of Clinical Sciences at Monash Health, Faculty of Medicine, Nursing and Health Sciences, Monash University, Melbourne, Australia; 2 Monash Centre for Health Research and Implementation, School of Public Health and Preventive Medicine, Faculty of Medicine, Nursing and Health Sciences, Monash University, Melbourne, Australia

## Abstract

Vitamin D is reported to have anti-inflammatory properties; however the effects of vitamin D supplementation on inflammation in patients with heart failure (HF) have not been established. We performed a systematic review and meta-analysis examining effects of vitamin D supplementation on inflammatory markers in patients with HF. MEDLINE, CINAHL, EMBASE, All EBM, and Clinical Trials registries were systematically searched for RCTs from inception to 25 January 2017. Two independent reviewers screened all full text articles (no date or language limits) for RCTs reporting effects of vitamin D supplementation (any form, route, duration, and co-supplementation) compared with placebo or usual care on inflammatory markers in patients with heart failure. Two reviewers assessed risk of bias and quality using the grading of recommendations, assessment, development, and evaluation approach. Seven studies met inclusion criteria and six had data available for pooling (n = 1012). In meta-analyses, vitamin D-supplemented groups had lower concentrations of tumor necrosis factor-alpha (TNF-α) at follow-up compared with controls (n = 380; p = 0.04). There were no differences in C-reactive protein (n = 231), interleukin (IL)-10 (n = 247) or IL-6 (n = 154) between vitamin D and control groups (all p > 0.05). Our findings suggest that vitamin D supplementation may have specific, but modest effects on inflammatory markers in HF.

## Introduction

Heart failure (HF) is a complex and increasingly common condition affecting 26 million people worldwide^1^. HF is associated with morbidity, loss of physical function, and a cascade of neuro-hormonal and peripheral muscle effects^2^. Although the pathophysiology of HF is not fully understood, increasing evidence suggests that the development and clinical course of HF is underscored by an inflammatory milieu including pro- and anti-inflammatory cytokines, adhesion molecules, and reactive oxygen species^3^. Therefore, strategies to reduce inflammation in patients with HF may reduce symptoms and improve overall health outcomes for these patients.

Vitamin D is reported to have anti-inflammatory and immune-modulating properties^4^, and a recently published randomized trial reported that vitamin D supplementation improved left ventricular structure and function in patients with HF^5^. A potential role for vitamin D in HF is supported by the widespread distribution of the vitamin D receptor (VDR) and metabolizing enzymes throughout the cardiovascular system, including in cardiac myocytes^6^. Absence of the VDR has resulted in cardiac remodeling and subsequent myocardial hypertrophy in mice^7^. Human epidemiological studies report that vitamin D deficiency [25-hydroxyvitamin D (25(OH)D) concentrations < 50 nmol/l] is common in individuals with HF and has been associated with reduced left ventricular ejection fraction (LVEF), increased natriuretic peptides, and increased mortality^8,9^. Increased concentrations of inflammatory markers in HF patients have also been associated with these same outcomes^10^. In contrast, vitamin D supplementation was recently shown to improve LVEF and reverse LV remodeling^5^, improve strength and balance, and reduce the risk of falls in patients with HF^11^. Use of vitamin D supplements may improve HF symptoms and outcomes via an anti-inflammatory pathway. However, the effect of vitamin D supplementation on inflammatory markers in patients with HF has not been established. We aimed to address this knowledge gap by performing a comprehensive systematic review and meta-analysis of all randomized controlled trials (RCTs) examining the effects of vitamin D supplementation on inflammatory markers in patients with HF.

## Research Design and Methods

This systematic review conforms to the Preferred Reporting Items for Systematic Reviews and Meta-analyses (PRISMA) guidelines (Appendix 1) and is part of a wider evidence synthesis of the effects of vitamin D on inflammation in multiple diseases. The methods for this work were specified *a priori* in a published protocol^12^, and a protocol for this meta-analysis was registered on PROSPERO (CRD:42016047753).

### Data Sources and Search Strategy

Studies were identified by systematically searching electronic databases using relevant search terms (Appendix 2) and pre-specified criteria, as outlined in our protocol^12^. Literature was searched from inception to 25 January 2017 for human studies, with no date or language limits. The search was conducted using the following electronic databases: MEDLINE; Medline in-process and other non-indexed citations; CINAHL; EMBASE; and All Evidence Based Medicine (EBM) Reviews. Additional studies were sought manually by searching the National Institute of Health Clinical Trials (https://clinicaltrials.gov/) and Australian New Zealand Clinical Trials (https://www.anzctr.org.au) registries, and via reference lists from relevant studies.

### Study screening and selection

**Figure 1:**
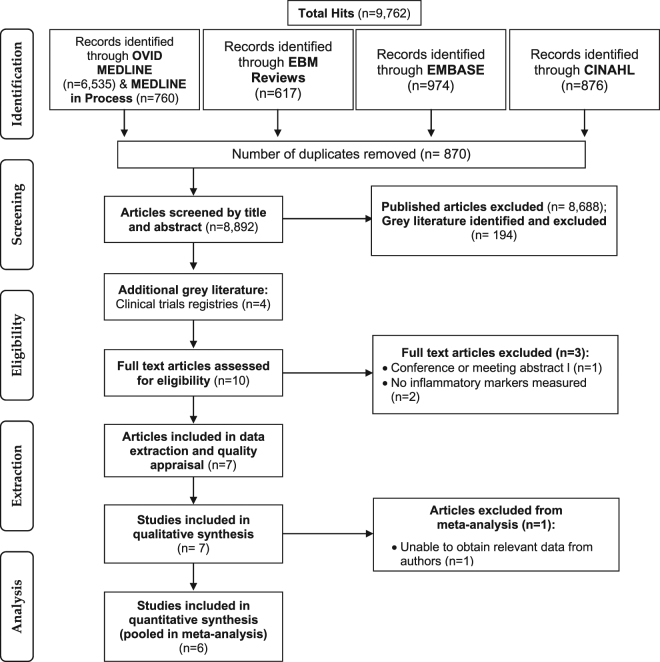
CONSORT diagram of the screening and selection process.

Selection criteria using the PICOS (Population, Intervention, Comparison, Outcomes, Study design) framework established *a priori* were used to determine eligibility of articles as previously reported^12^. Eligibility criteria are outlined in Supplementary Table 1. Briefly, included studies were RCTs in any population with diagnosed HF, where the intervention was vitamin D supplementation provided in any form, dose, or route, compared to placebo or usual care, and with inflammatory marker outcomes. Studies conducted in participants without diagnosed HF or which did not use vitamin D supplementation were excluded. The selection process is outlined in Fig. 1. Two independent reviewers (AJR and AM) examined full-text articles to confirm eligibility, and consensus was resolved by discussion or referred to a third reviewer (BdC).

### Data extraction

Two independent reviewers (AJR, AM) performed data extraction using a specifically developed template, which included study author, year, and design; sample size and demographics; randomization strategy; vitamin D regimen/s and co-intervention/s and type of control/comparator used; outcome definition and assessment; mean values of outcomes and their standard deviations or confidence intervals, point estimates, and measures of variability; frequency counts for dichotomous variables; and intention-to-treat analysis. Corresponding authors of included trials where required data were not presented were contacted to provide de-identified data (aggregated effect measures) for the purpose of meta-analysis.

### Risk of bias and quality appraisal

Risk of bias was assessed at the study-level by two independent reviewers (AJR and AM) using a critical appraisal template (Appendix 3) with pre-specified criteria^12^. Individual quality items were examined using a descriptive component approach as previously described^12^ with assessment of study design aspects (Appendix 3). Using this approach, each study was assigned a risk of bias rating (high, moderate, low, or insufficient information).

Quality of the evidence was assessed at the outcome-level using the grading of recommendations, assessment, development and evaluation (GRADE) approach^13^. Two independent reviewers (AJR and AM) graded quality of the evidence based on risk of bias, imprecision (upper or lower limit of 95% CI is > 0.5), inconsistency (heterogeneity), indirectness (heterogeneous participants, outcomes, or interventions), and suspicion of publication bias. Interpretation of the grading scores are presented in Supplementary Table 2. Disagreements were resolved by consensus.

### Data synthesis and analysis

Data are presented in summary tables and in brief narrative form to describe the included studies. For meta-analysis, aggregate effect measures at the end of the intervention period were pooled into random-effects models and standardized mean differences (SMD) with 95% confidence intervals (95% CI) were computed since studies used different methods/assays and reported different inflammatory marker concentrations. Statistical heterogeneity was assessed using the *I*^2^ test, where *I*^2^ > 50% indicated moderate to high heterogeneity for which sensitivity analyses were applied. In sensitivity analyses, studies deemed as high or moderate risk of bias and/or which contributed to heterogeneity or had different participant characteristics (eg: studies in infants versus adults) were omitted to examine their effects on the results. For meta-analyses of more than two studies, visual inspection of funnel plots and Egger^14^ and Begg^15^ statistical tests were used to determine small study effects and potential publication bias. Meta-analyses were performed in Review Manager V.5.3.5 and publication bias was assessed using Comprehensive Meta-analysis Software V.3. *P*-values < 0.05 were considered statistically significant.

### Data availability

The datasets generated and analysed during the current study are available from the corresponding author on reasonable request.

## Results

Outcomes of the search and screening process are presented in Fig. 1. Initial database searches for all RCTs of vitamin D supplementation (Appendix 2) yielded 9,762 records, of which 870 were duplicates. Abstracts and titles were screened for the remaining 8,892 records (including 194 grey literature records). Six records which were in HF patients with inflammatory marker outcomes were eligible for full-text assessment (Fig. 1). An additional four records were identified by manual searches and via clinical trials registries, totaling 10 records which were eligible for full-text review. Of these 10 studies, three were excluded with reasons outlined in Fig. 1, thus seven studies^16–22^ met the inclusion criteria for qualitative synthesis.

### Study characteristics

Descriptive data of the included studies are summarized in Table 1 and detailed in Supplementary Tables 3 and 4. All studies were in English-language and of parallel design. Study durations ranged from 6 weeks^19^ to 12 months^17^, with a mean duration of 7 months across the studies. Most studies enrolled older adults (age > 50 years), with one study investigating HF in infants^20^ (Table 1).

**Table 1:** Characteristics of studies included in systematic review of effects of vitamin D supplementation on inflammation in patients with heart failure.

Author, Year, Country	Design; Setting	N (n)*	Participants; (% male)	Intervention and Control arms	Frequency/duration	Baseline age (y); BMI (kg/m^2^); and HF duration (months)		Baseline 25(OH)D (nmol/l)	Primary outcome/s	Biomarkers	Pooled
Boxer, 2014, USA	Parallel RCT; Academic HF and general cardiology practices	64 (64);	Adults > 50 y old with HF; (51% male)	**I:** 50,000 IU oral VD3 + 800 mg Ca; **P:** placebo + 800 mg Ca	Weekly 6 months	**Age=**	**I:** 65.8 ± 10.6 **P:**66.0 ± 10.4	**I:** 47.7 ± 7.5 **P:** 44.4 ± 22.5	RAAS	CRP	Yes
						**BMI=**	**I:** 34.8 ± 7.2 **P:** 31.3 ± 6.9				
						**Duration**=	**NR**				
McKeag, 2014, Northern Ireland	Parallel RCT; Hospital-based HF clinics	74 (74)	Adults with stable HF; (81% male)	**I:** 1,000 IU oral VD3 + 400 IU VD2; **P:** placebo (lactose)	Daily 12 months	**Age=**	**I:** 65.8 ± 9.4 **P:** 62.7 ± 9.0	**I:** 38.7 ± 13.8 **P:** 38.6 ± 23.7	LVEF, QoL, 6 min walk distance	IL-6, IL-10, TNF-α, CRP	Yes
						**BMI=**	**I:** 29.5 ± 2.4 **P:** 29.9 ± 5.9				
						**Duration**=	NR				
Schleithoff, 2006, Germany	Parallel RCT; Heart and Diabetes Centre	123 (93)	Adults with congestive HF; (83% male)	**I:** 2,000 IU oral VD3 + 500 mg Ca; **P:** Miglyol oil + 500 mg Ca	Daily 9 months	**Age=**	**I:** 57 (53, 63) **P:**54 (50, 62)	**I:** 35.9 (28.7, 55.2) **P:** 38.2 (31.7, 56.9)	Biochemical markers, LVEF, VO2 max	TNF-α, CRP, IL-6, IL-10	Yes
						**BMI=**	**I:** 26 (23.9, 29) **P:** 25.4 (24.3, 28.4)				
						**Duration**=	NR				
Schroten, 2013, Holland	Parallel RCT; Outpatient clinic	101 (94)	Adults chronic HF on optimal medical therapy; (93% male)	**I:** 2,000 IU oral VD3; **P:** NR	Daily 6 weeks	**Age=**	**I:** 63.5 ± 11.1 **P:** 64.0 ± 9.0	**I:** 46 (39, 63) **P:** 48 (38,61)	Plasma renin activity	Ngal, FGF-23	No^†^
						**BMI=**	NR				
						**Duration**=	**I:** 62 (34, 102) **P:** 61 (29, 133)				
Shedeed, 2012, Egypt	Parallel RCT; Teaching hospital cardiology unit of paediatric department	80 (80)	Infants with congestive HF; (61% male)	**I:** 1,000 IU oral VD3; **P:** placebo (dH2O)	Daily 3 months	**Age=**	**I:** 10.3 ± 4.6^a^ **P:** 11.2 ± 3.5^a^	**I:** 33.5 ± 5.5 **P:** 34.9 ± 6.2	RAAS	IL-10, IL-6, TNF-α	Yes
						**BMI=**	N/A				
						**Duration**=	**I:** 5.39 ± 2.1 **P:** 5.11 ± 1.9				
Witham, 2010, UK	Parallel RCT; Primary and secondary care facilities	105 (84)	Older adults with chronic HF and low vitamin D (<50nmol/L); (66% male)	**I:** 100,000 IU oral VD2; **P:** NR	bolus doses quarterly (x3) 9 months	**Age=**	**I:** 78.8 ± 5.6 **P:** 80.6 ± 5.7	**I:** 20.5 ± 8.9 **P:** 23.7 ± 10.0	6 min walk, TUG, RAAS, BP	TNF-α	Yes
						**BMI=**	**I:** 27.2 ± 5.1 **P:** 27.3 ± 4.5				
						**Duration**=	NR				
Witte, 2005, UK	Parallel RCT; Community-based HF unit	28 (28)	Older >70 y adults with chronic HF due to ischemia; (NR% male)	**I:** 400 IU oral VD (type NR) + 250 mg Ca; **P:** NR	Daily 9 months	**Age=**	**I:** 74.2 ± 2.8 **P:** 75.5 ± 3.5	**I:** NR **P:** NR	LVEF, QoL, inflammatory cytokines	TNF-α, IL-6, TNFR-1, TNFR-2	Yes
						**BMI=**	**I:** 27.8 ± 2.4 **P:** 26.4 ± 3.5				
						**Duration**=	NR				

Data presented as mean ± standard deviation or median (interquartile range), unless otherwise specified. *N (n) = Number of participants randomized (number analyzed); ^†^Unable to obtain all or some relevant outcome data from authors; ^a^data represents months. Abbreviations: **HF**, heart failure; **RCT**, randomized controlled trial; **BMI**, body mass index; **VD3**, vitamin D3/cholecalciferol; **VD2**, vitamin D2/ ergocalciferol; **Ca**, calcium; **IU**, international units; **I**, intervention group; **P**, placebo/control group; **BP**, blood pressure; **RAAS**, renin-angiotensin-aldosteron system; **LVEF**, left ventricular ejection fraction; **QoL**, quality of life; **VO2 max**, maximum volume of oxygen; **TUG**, Timed Up and Go test; **CRP**, C-reactive protein; **IL**, interleukin; **TNF- α**, tumor necrosis factor-alpha; **Ngal**, neutrophil gelatinase-associated lipocalin; **FGF-23**, fibroblast growth factor-23; **TNFR-1/-2**, tumor necrosis factor receptor-1/-2; **NR**, not reported; **N/A**, not applicable; **mo**, months; **y**, year.

### Participant Characteristics

In studies of adult patients (n = 6 RCTs), the mean age of participants ranged from 62.7 to 80.6 years, while Schleithoff *et al*.^18^ reported a median age of 57 and 54 years in vitamin D and placebo groups, respectively (Table 1). Males made up >50% of participants in the 6 studies reporting gender distribution. Mean/median baseline body mass index (BMI) as reported in five studies ranged from 25.4 to 34.8 kg/m^2^. Mean and median baseline 25(OH)D concentrations ranged between 20.5–47.7 nmol/l and 35.9–48 nmol/l, respectively, as reported in six studies (Table 1). Only one study^21^ explicitly excluded non-vitamin D-deficient participants [25(OH)D > 50 nmol/l]. Two studies reported HF duration (Table 1), while smoking status was reported in only three studies (Supplementary Table 4). Recruitment of participants was based on severity of symptoms determined by New York Heart Association (NYHA) classifications ≥II^16,18,21^; LVEF ≤ 35%, ≤40% or <45%^19,20,22^; or both NYHA class II-III and LVEF ≤ 45%^17^. Only one study^16^ reported ethnicity, where the proportion of African-Americans was approximately 63% of enrolled participants.

### Intervention Characteristics

Oral cholecalciferol supplementation was used in five studies, with doses ranging from 1,000–2,000 IU daily^17–20^ to a weekly dose of 50,000 IU^16^. Of these five studies, two co-supplemented oral cholecalciferol with calcium^16,18^ and another supplemented 1,000 IU oral cholecalciferol in addition to 400 IU ergocalciferol daily as part of the Forceval© multivitamin supplement^17^. In the remaining two studies which did not use cholecalciferol^21,22^, one used 100,000 IU of oral ergocalciferol administered three times over nine months^21^ and the other did not specify the type of vitamin D used but stated an oral dose of 400 IU daily as part of a micronutrient^22^.

### Outcome Measures

Most studies reported LVEF^17,18,22^, or renin-angiotensin-aldosterone-system (RAAS) activity^16,19–22^ as primary outcomes. Various inflammatory markers were examined (Table 1), the most common of which was TNF-α, measured in 5 of the 7 RCTs. Other commonly measured markers included C-reactive protein (CRP) (n = 3 RCTs), interleukin (IL)-6 (n = 4 RCTs), and IL-10 (n = 3 RCTs) (Table 1).

### Risk of Bias assessment

Results of the risk of bias assessment are presented in Supplementary Table 5. All studies were double-blinded, except one which only employed single blinding of participants^19^. All studies reported dropout rates; however only three performed intention-to-treat analyses^16,17,20^. Selective reporting was evident in three studies^16,19,22^. Overall, most studies were rated as having high (n = 3) or moderate (n = 2) risk of bias, with three studies having low risk of bias.

### Meta-analyses and sensitivity analyses

One of the seven studies^19^ did not have available data for pooling and was excluded from meta-analysis. Data from the remaining six studies were pooled to examine differences in inflammatory markers between vitamin D and placebo groups at follow-up. Markers such as fibroblast growth factor-23 (FGF-23) and TNF-receptors had available data from single studies, and are therefore included in the descriptive analysis component.

**Figure 2:**
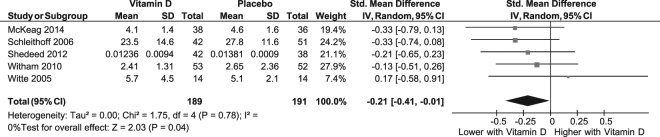
Forest plot showing results of a meta-analysis of the effects of vitamin D supplementation on tumor necrosis factor- *alpha*. Data are reported as SMDs with 95% CIs.

Pooling of five RCTs (n = 380)^17,18,20–22^ showed a significant difference in TNF-α concentrations between vitamin D and placebo groups at follow up [SMD (95%CI) = −0.21 (−0.41, −0.01); p = 0.04; *I*^2^ = 0%; *P*_*het*_ = 0.8] (Fig. 2). In a sensitivity analysis, excluding the study in infants^20^ attenuated the difference between vitamin D and placebo groups [SMD (95%CI): −0.21 (−0.44, −0.02); p = 0.07; *I*^2^ = 0%; *P*_*het*_ = 0.6]. However, in a further sensitivity analysis excluding the study in infants^20^ and including only low risk of bias studies^17,18,21^, there was a significant difference in follow-up TNF-α concentrations between vitamin D and placebo groups [SMD (95%CI): −0.25 (−0.49, −0.01), p = 0.04; *I*^2^ = 0%; *P*_*het*_ = 0.7].

**Figure 3:**
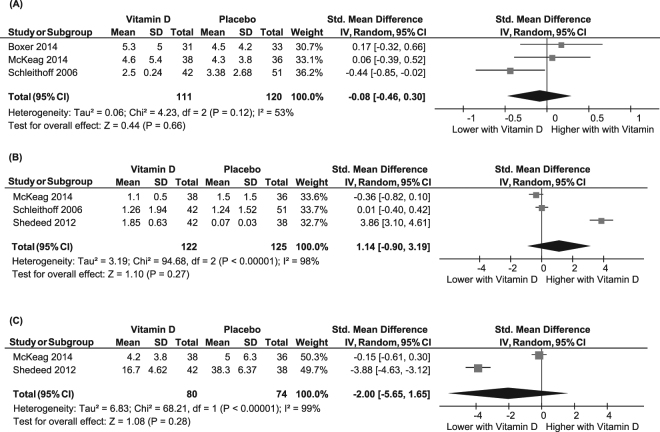
Forest plot showing results of a meta-analysis of the effects of vitamin D supplementation on C-reactive protein (**A**), interleukin-10 (**B**) and interleukin-6 (**C**). Data are reported as SMDs with 95% CIs.

Pooling of data from three studies (n = 231)^16–18^ showed no significant difference between vitamin D and placebo groups in follow up CRP concentrations, with moderate heterogeneity [SMD (95%CI): −0.08 (−0.46, 0.30); p = 0.7; *I*^2^ = 53%; *P*_*het*_ = 0.1] (Fig. 3A). Results remained non-significant in a sensitivity analysis limited to only low risk of bias studies^17,18^ [SMD (95%CI): −0.20 (−0.69, 0.29); p = 0.4; *I*^2^ = 61%; *P*_*het*_ = 0.1].

In pooled analysis of data from three studies (n = 247)^17,18,20^, there were no significant differences in IL-10 concentrations between vitamin D and placebo groups at follow up [SMD (95%CI): 1.14 (-0.90, 3.19); p = 0.3; *I*^2^ = 98%; *P*_*het*_ < 0.001], with significant heterogeneity (Fig. 3B). In a sensitivity analysis excluding the study in infants ^20^ which was also responsible for the significant heterogeneity, differences in IL-10 between groups remained non-significant^17,18^ [SMD (95%CI): −0.16 (−0.52, 0.20); p = 0.4; *I*^2^ = 28%; *P*_*het*_ = 0.4]. Both studies in this sensitivity analysis were deemed low risk of bias^17,18^.

Similarly, pooled analysis of two studies (n = 154)^17,20^ showed lower concentrations of IL-6 in the vitamin D group; however this was not statistically significant and heterogeneity was observed (SMD (95%CI): −2.00 (−5.65, 1.65); p = 0.3; *I*^2^ = 99%; *P*_*het*_ < 0.001) (Fig. 3C). Given only two studies were included, further sensitivity analyses to explore or exclude sources of bias or heterogeneity were not possible.

### Subgroup Analyses

Study and sample characteristics thought to be clinically relevant to the outcomes were assessed in subgroup analyses for TNF-α, CRP, and IL-10 as these markers had a sufficient number of studies (≥3 RCTs). Studies were stratified by dose (≤1000, or >1000 IU) and duration (≤6, or >6 months) of vitamin D supplementation and whether vitamin D was co-supplemented with calcium (>100 mg daily versus ≤100 mg or no calcium). There were no differences in TNF-α, CRP, or IL-10 between any of the subgroups (all p > 0.05; data not shown). Stratified analyses by baseline vitamin D status, BMI, or age were not possible since all studies had similar mean baseline 25(OH)D concentrations (<50 nmol/l) and mean ages and BMIs (>50 years and >25 kg/m2, respectively, except for the study in infants which was excluded in sensitivity analysis).

### Descriptive Analyses

In a study that was excluded from meta-analysis due to unavailable data^19^, 2000 IU daily of cholecalciferol for 6 weeks had no effect on FGF-23 or neutrophil gelatinase-associated lipocalin in patients with HF. Another study^22^ measured IL-6 (data not available for meta-analysis) as well as TNF receptors 1 and 2 and found no effects after supplementation with 400 IU of oral vitamin D (type not specified) daily for 9 months.

### GRADE assessment and Publication Bias

Based on visual inspection of funnel plots (Supplementary Figure 1), as well as Egger^14^ and Begg^15^ statistical tests (Supplementary Table 6), we found no evidence of publication bias for TNF-α, CRP, or IL10. Studies reporting on IL-6 could not be assessed for publication bias due to the small number of studies (n = 2 RCTs).

An evaluation of the quality of evidence using the GRADE approach^13^ is presented in Supplementary Table 7. For TNF-α, the quality of evidence was high since most studies had low to moderate risk of bias with low statistical and clinical heterogeneity and narrow CIs. Moreover, although the effect for TNF-α was considered small (SMD < 0.5), it persisted in a sensitivity analysis including only low risk of bias studies. For CRP, quality of the evidence was deemed moderate due to imprecision (wide CI) and moderate heterogeneity. For IL6 and IL10, the evidence was deemed low quality due to high risk of bias, imprecision, and inconsistency (*I*^2^ > 90% for heterogeneity), as well as having small numbers of studies and potential reporting bias (Supplementary Table 7).

## Discussion

### Summary of Findings

In this systematic review and meta-analysis of RCTs in patients with HF, we found that vitamin D-supplemented groups had lower TNF-α concentrations, which persisted in a sensitivity analysis including only low risk of bias studies. There were no differences between vitamin D and placebo groups in CRP, IL-10 or IL-6. Our findings suggest that vitamin D may have specific, but modest effects on inflammation in patients with HF.

### Summary of Previous Evidence

Vitamin D is a pleiotropic steroid hormone, which elicits its functions by acting through the vitamin D receptor (VDR)^23^. The VDR is present in many cell types including cardiac myocytes^23^. In animal studies, mice defective in 1α-hydroxylase (the enzyme which converts inactive vitamin D to its active form) had altered calcium handling which led to exaggerated cardiac dysfunction consistent with HF in humans^24^. Additionally, these mice exhibited increased expression of the pro-inflammatory cytokines TNF-α and monocyte chemoattractant protein-1 (MCP-1), however a normal phenotype was restored upon supplementation with vitamin D, highlighting a potential role for vitamin D in HF pathogenesis^24^.

Observational studies in humans support pre-clinical findings. Low serum 25(OH)D concentrations have consistently been observed in patients with HF, and have been associated with HF severity^25^, and with inflammatory markers in HF^26^. Given these findings, RCTs sought to explore the clinical utility of vitamin D supplementation in improving inflammatory profiles in HF. As shown in our meta-analysis, vitamin D treatment may reduce circulating TNF-α concentrations; however, the observed effect was relatively small, and no study that contributed to this analysis^17,18,20–22^ showed concomitant improvements in clinical or laboratory markers of HF such as LVEF or natriuretic peptides. Furthermore, a meta-analysis of seven RCTs in patients with HF^27^ found that vitamin D supplementation did not improve clinical symptoms including LVEF, 6-minute walk test, and natriuretic peptide concentrations. It is therefore unclear if resolution of systemic inflammation can improve cardiac physiology and outcomes in patients with HF.

Our results support the findings of a previous meta-analysis by Jiang *et al*.^27^, although our meta-analysis included a greater number of studies and markers, and a larger overall sample. Jiang *et al*.^27^ reported no effects of vitamin D supplementation on IL-10 concentrations; however follow-up TNF-α and CRP concentrations were lower in vitamin D-supplemented groups compared with controls. Importantly, results for TNF-α in the meta-analysis by Jiang *et al*.^27^ were based on pooled analysis of three RCTs (n = 257), while only two RCTs were pooled for CRP (n = 185). Here, we add to existing evidence by showing that the effect for TNF-α persisted in a larger meta-analysis of five RCTs totaling 380 patients, and importantly, that there was no effect on CRP when data from all three studies which measured CRP were pooled (n = 231). The present study also adds to current evidence by providing additional analysis of two RCTs (n = 154) reporting on IL-6, where we observed lower concentrations in the vitamin D group compared with placebo at follow-up, though this did not reach statistical significance. The observed effects, or lack thereof, persisted in sensitivity analyses of only low risk of bias studies. This adds robustness to our findings since sensitivity analyses were not performed in the previous meta-analysis^27^.

### Limitations of the Evidence

Overall, the current literature is limited. Most studies had small samples, with <100 participants in all but one study (n = 101)^19^. Quality of the evidence across studies was low or moderate for most markers, and only the evidence for TNF-α was deemed high quality. Moreover, no study accounted for seasonal variation or sunlight exposure, and/or physical activity, which may potentially influence vitamin D levels, as well as body composition since vitamin D is fat-soluble and sequestered in adipose tissue^28^. Smoking status and HF duration were also not reported in several studies - factors which may influence inflammatory status in these patients. Finally, only one study actively recruited patients who were vitamin D-deficient at baseline^21^. Increasingly, it has been shown that beneficial effects of vitamin D supplementation are only observed when provided to vitamin D-deficient individuals^29^. Thus, future trials recruiting only vitamin D-deficient individuals may strengthen the evidence base, as could subgroup analyses comparing inflammatory marker profiles in vitamin D deficient versus replete individuals at follow-up. We were unable to perform subgroup analyses by baseline vitamin D status, or by studies that achieved adequate vitamin D status at follow-up due to the small number of studies and lack of reporting of follow-up vitamin D levels in most studies.

### Study Strengths and Limitations

Our study has some limitations. First, although most studies had low to moderate risk of bias, some high risk of bias studies included in the main analysis may have influenced the results. However, we performed sensitivity analyses where possible to account for the effects of high risk of bias studies. Moreover, randomization, blinding, and the use of a control group were considered the most important aspects in our meta-analysis, and most studies satisfied these criteria. Second, the studies identified were heterogeneous in terms of type of vitamin D used (cholecalciferol or ergocalciferol) and the dosing protocols, which may introduce some confounding to our analysis. We also could not ascertain whether biomarker values reported in each paper were derived from normal or skewed distributions, thus results should be interpreted with caution. Given the limited number of studies, we were unable to conduct subgroup analyses for certain study design aspects such as comparing daily versus monthly doses, and we were unable to perform meta-regression to account for other factors such as BMI or baseline vitamin D status of participants. Third, publication bias cannot be ruled out for markers with few studies or where we were unable to obtain all necessary data from authors. Finally, whilst our meta-analysis suggests that vitamin D may reduce TNF-α concentrations, the observed effect was small, and it remains unclear whether such effects would translate into improved health outcomes in patients with HF.

Nevertheless, we included only RCTs, the gold-standard for establishing causality. We applied rigorous international gold-standard methodology and conformed to international reporting standards with a protocol published *a priori* to ensure transparency. Our search strategy was comprehensive and included non-English language publications and grey literature. We sought data directly from authors in order to provide a more comprehensive meta-analysis with inclusion of further data and more inflammatory markers than the previous meta-analysis^27^. This meant that we were able to perform sensitivity analyses for some markers including TNF-α, not previously performed. These factors add robustness to our study and enable us to provide a comprehensive and up-to-date synthesis of current evidence of the effects of vitamin D supplementation on inflammatory markers in patients with HF.

## Conclusions

In conclusion, we showed that vitamin D-supplemented groups had a small but significantly lower TNF-α concentration at follow-up compared with placebo. Although vitamin D may not be effective as a sole treatment to improve inflammation or HF outcomes, it may be beneficial as an adjunct to existing therapies in vitamin D-deficient patients with HF. However, further large-scale, well-designed trials including vitamin D-deficient participants and measuring both inflammatory markers and long-term clinical HF endpoints are needed to determine if vitamin D supplementation can reduce inflammatory markers and improve health outcomes for patients with HF.

## Electronic supplementary material

Supplementary Information

## References

[1] Ambrosy AP (2014). The global health and economic burden of hospitalizations for heart failure: lessons learned from hospitalized heart failure registries. J Am Coll Cardiol.

[2] Mentz RJ, O’Connor CM (2016). Pathophysiology and clinical evaluation of acute heart failure. Nat Rev Cardiol.

[3] Matsumori A, Yamada T, Suzuki H, Matoba Y, Sasayama S (1994). Increased circulating cytokines in patients with myocarditis and cardiomyopathy. British Heart Journal.

[4] Gysemans CA (2005). 1,25-Dihydroxyvitamin D3 modulates expression of chemokines and cytokines in pancreatic islets: implications for prevention of diabetes in nonobese diabetic mice. Endocrinology.

[5] Witte KK (2016). Effects of Vitamin D on Cardiac Function in Patients With Chronic HF: The VINDICATE Study. Journal of the American College of Cardiology.

[6] Meems LM, van der Harst P, van Gilst WH, de Boer RA (2011). Vitamin D biology in heart failure: molecular mechanisms and systematic review. Current drug targets.

[7] Chen S (2008). Expression of the vitamin d receptor is increased in the hypertrophic heart. Hypertension.

[8] Liu LC (2011). Vitamin D status and outcomes in heart failure patients. European journal of heart failure.

[9] Zittermann A (2003). Low vitamin D status: a contributing factor in the pathogenesis of congestive heart failure?. J Am Coll Cardiol.

[10] Tromp, J. *et al*. Biomarker Profiles in Heart Failure Patients With Preserved and Reduced Ejection Fraction. *Journal of the American Heart Association* **6**, 10.1161/jaha.116.003989 (2017). 10.1161/JAHA.116.003989 PMC5532986 28360225

[11] Amin A (2013). Can Vitamin D Supplementation Improve the Severity of Congestive Heart Failure?. Congestive Heart Failure.

[12] Mousa, A., Misso, M., Teede, H., Scragg, R. & de Courten, B. Effect of vitamin D supplementation on inflammation: protocol for a systematic review. *BMJ open* **6** (2016). 10.1136/bmjopen-2015-010804 PMC4823456 27048637

[13] Atkins D (2004). Grading quality of evidence and strength of recommendations. Bmj.

[14] Egger M, Davey Smith G, Schneider M, Minder C (1997). Bias in meta-analysis detected by a simple, graphical test. Bmj.

[15] Begg CB, Mazumdar M (1994). Operating characteristics of a rank correlation test for publication bias. Biometrics.

[16] Boxer RS (2014). The effect of vitamin d on aldosterone and health status in patients with heart failure. J Card Fail.

[17] McKeag NA (2014). The effect of multiple micronutrient supplementation on left ventricular ejection fraction in patients with chronic stable heart failure: A randomized, placebo-controlled trial. JACC: Heart Failure.

[18] Schleithoff SS (2006). Vitamin D supplementation improves cytokine profiles in patients with congestive heart failure: a double-blind, randomized, placebo-controlled trial. Am J Clin Nutr.

[19] Schroten NF (2013). Short-term vitamin D3 supplementation lowers plasma renin activity in patients with stable chronic heart failure: an open-label, blinded end point, randomized prospective trial (VitD-CHF trial). Am Heart J.

[20] Shedeed SA (2012). Vitamin D supplementation in infants with chronic congestive heart failure. Pediatr Cardiol.

[21] Witham MD, Crighton LJ, Gillespie ND, Struthers AD, McMurdo ME (2010). The effects of vitamin D supplementation on physical function and quality of life in older patients with heart failure: a randomized controlled trial. Circ.

[22] Witte KK (2005). The effect of micronutrient supplementation on quality-of-life and left ventricular function in elderly patients with chronic heart failure. Eur Heart J.

[23] Anagnostis P, Athyros VG, Adamidou F, Florentin M, Karagiannis A (2010). Vitamin D and cardiovascular disease: a novel agent for reducing cardiovascular risk?. Current vascular pharmacology.

[24] Choudhury S (2014). Abnormal calcium handling and exaggerated cardiac dysfunction in mice with defective vitamin d signaling. PloS one.

[25] Belen E (2014). Clinical staging in chronic heart failure associated with low vitamin D and elevated parathormone levels. Acta cardiologica.

[26] Boxer RS, Dauser DA, Walsh SJ, Hager WD, Kenny AM (2008). The association between vitamin D and inflammation with the 6-minute walk and frailty in patients with heart failure. Journal of the American Geriatrics Society.

[27] Jiang WL (2016). Vitamin D Supplementation in the Treatment of Chronic Heart Failure: A Meta-analysis of Randomized Controlled Trials. Clin Cardiol.

[28] Mutt SJ, Hypponen E, Saarnio J, Jarvelin MR, Herzig KH (2014). Vitamin D and adipose tissue-more than storage. Frontiers in physiology.

[29] von Hurst PR, Stonehouse W, Coad J (2010). Vitamin D supplementation reduces insulin resistance in South Asian women living in New Zealand who are insulin resistant and vitamin D deficient - a randomised, placebo-controlled trial. The British journal of nutrition.

