# Supplementary material for: Effects of vitamin D supplementation on inflammatory markers in heart failure: a systematic review and meta-analysis of randomized controlled trials
Source: Sci Rep. 2018 Jan 18;8:1169. doi: 10.1038/s41598-018-19708-0 (PMC5773527; doi:10.1038/s41598-018-19708-0)
Supplement: Supplementary file 1 — Supplementary Information [file 41598_2018_19708_MOESM1_ESM.pdf]

Title: Effects of vitamin D supplementation on inflammatory markers in heart failure: a systematic review and meta-analysis of randomized controlled trials
Authors: Alexander J. Rodriguez, Aya Mousa, Peter R. Ebeling, David Scott, Barbora de Courten

SUPPLEMENTAL MATERIAL

Supplementary Table 1. Eligibility criteria for study selection (PICOS)

	**Participants (P)**	**Intervention (I)**	**Comparison (C)**	**Outcomes (O)**
**Inclusion**	Males and female patients with heart failure diagnosed by NYHA class, LVEF, or brain natriuretic peptide, on any treatment regimen and for any duration, of any age, ethnicity, socio-economic status, geographic area, co-morbidity or pregnancy status	Any type of vitamin D supplementation (D2; D3; calcitriol; analoges) administered in any form (oral, intravenous, or intramuscular) alone or combined with other intervention/s, of any dosage, and for any duration	Placebo or usual care; any other non-pharmacological interventions or pharmacological interventions	Inflammatory biomarkers including but not limited to: all interleukins, all TNFα, TGF-β1, CRP, MCP-1, IFNγ, NFκB, MIF, fibrinogen, adipokines: leptin, resistin, visfatin, adiponectin, omentin
**Exclusion**	Studies in participants without diagnosed heart failure	Studies without vitamin D supplementation	Studies with no control/comparator group	Studies with no inflammatory marker outcomes measured
**Study type (S)**		Systematic reviews of RCTs and RCTs in humans		
**Language**		No limit		
**Year of publication**		No limit		

Abbreviations: NYHA, New York Heart Association; **LVEF**, left ventricular ejection fraction; **TNFα**, tumor necrosis factor-alpha; **TGF-β1**, transforming growth factor-beta 1, **CRP**, C-reactive protein; **MCP-1**, monocyte chemoattractant protein-1; **IFN-γ**, interferon-gamma; **NFκB**, nuclear factor kappa B; **MIF**, macrophage migration inhibitory factor; **RCTs**, randomized controlled trials.

Supplementary Table 2. Grading the quality of the evidence (adapted from GRADE Working Group, 2004)

**Strength of Evidence**	**Interpretation**
**High quality**	Very confident in the estimate of the effect and further research is very unlikely to change our confidence.
**Moderate quality**	Moderately confident in the estimate of the effect, but further research may have an important impact on our confidence and may change the estimate.
**Low quality**	Somewhat confident in the estimate of the effect, but further research is very likely to have an important impact on our confidence and will likely change the estimate.
**Very low quality**	Very little confidence in the estimate of the effect as it is very uncertain.

Supplementary Table 3. Study and Intervention Characteristics

Study details	*n* (analyzed)	Intervention and Control arms	Total VD Dose (IU)	Frequency/ Duration	Route	Participant Characteristics	Baseline 25(OH)D (nmol/l)	Primary outcome	Biomarkers
Boxer 2014, USA	64 (64)	**I:** 50,000 IU oral VD3 + 800 mg Ca; **P:** placebo + 800 mg Ca	50,000	Weekly 6 months	Oral Capsule	>50y males and females with HF	**I:** 47.7 ± 7.5 **P:** 44.4 ± 22.5	RAAS	CRP
McKeag 2014, Northern Ireland	74 (74)	**I:** 1,000 IU oral VD3 + 400 IU VD2; **P:** placebo (lactose)	1000 + 400	Daily 12 months	Oral Capsule	Adults with stable HF	**I:** 38.7 ± 13.8 **P:** 38.6 ± 23.7	LVEF, QoL, 6min walk distance	IL-6, IL-10, TNF-α, CRP
Schleithoff 2006, Germany	123 (93)	**I:** 2,000 IU oral VD3+ 500 mg Ca; **P:** Miglyol oil + 500 mg Ca	2000	Daily 9 months	Oral Capsule	Adults with congestive HF	**I:** 35.9 (28.7,55.2) **P:** 38.2 (31.7,56.9)	Biochemical markers, LVEF, VO2 max	TNF-α, CRP, IL-10
Schroten 2013, Holland	101 (94)	**I:** 2,000 IU oral VD3; **P:** NR	2000	Daily 6 weeks	Oral Capsule	Adults chronic HF on optimal medical therapy	**I:** 46 (39, 63) **P:** 48 (38,61)	Plasma renin activity	Ngal, FGF-23
Shedeed 2012, Egypt	80 (80)	**I:** 1,000 IU oral VD3; **P:** placebo (dH2O)	1000	Daily 3 months	Oral Oil drop	Infants with congestive heart failure	**I:** 33.5 ± 5.5 **P:** 34.9 ± 6.2	RAAS	IL-10, IL-6, TNF-α
Witham 2010, UK	105 (84)	**I:** 100,000 IU oral VD2; **P:** NR	100,000	3 doses (quarterly for 9 months)	Oral Capsule	Older adults with HF with low vitamin D (<50nmol/L)	**I:** 20.5 ± 8.9 **P:** 23.7 ± 10.0	6min walk, TUG, RAAS, BP	TNF-α
Witte 2005, UK	28 (28)	**I:** 400 IU oral VD (type NR) + 250 mg Ca; **P:** NR	400	Daily 9 months	Oral Capsule	Older >70y adults with HF due to ischemia	NR	LVEF, QoL, inflammatory cytokines	TNF-α, IL-6, TNFR-1, TNFR-2

Abbreviations: **HF**, heart failure; **RCT**, randomized controlled trial; **BMI**, body mass index; **VD3**, vitamin D3/cholecalciferol; **VD2**, vitamin D2/ ergocalciferol; **Ca**, calcium; **IU**, international units; **I**, intervention group; **P**, placebo/control group; **BP**, blood pressure; **RAAS**, renin-angiotensin-aldosteron system; **LVEF**, left ventricular ejection fraction; **QoL**, quality of life; **VO2 max**, maximum volume of oxygen; **TUG**, Timed Up and Go test; **NT-proBNP**, N-terminal pro B-type natriuretic peptide; **CRP**, C-reactive protein; **IL**, interleukin; **TNF- α**, tumor necrosis factor-alpha; **8isoPGF2a**, 8-isoprotaglandin F2a ; **Ngal**, neutrophil gelatinase-associated lipocalin; **FGF-23**, fibroblast growth factor-23; **TNFR-1/-2**, tumor necrosis factor receptor-1/-2; **NR**, not reported; **N/A**, not applicable; **mo**, months; **y**, years.

Supplementary Table 4. Baseline participant characteristics and follow up biochemical analyses:

Study	*n*	Age (years)	Males *n* (%)	BMI (kg/m^2)	HF duration (months)	Current Smokers *n* (%)	Follow Up 25(OH)D (nmol/l)	Follow Up CRP (mg/L)	Follow Up TNF-a (pg/ml)	Follow Up IL-6 (pg/ml)	Follow Up IL10 (pg/ml)	Follow Up FGF-23 (RU/mL)
Boxer	**I:** 31<br>**P:** 33	**I:** 65.8 ± 10.6<br>**P:** 66.0 ±10.4	**I:** 15 (48)<br>**P:** 18 (54)	**I:** 34.8 ± 7.2<br>**P:** 31.3 ± 6.9	NR		NR	**I:** 5.3 ± 5.0<br>**P:**4.5 ± 4.2				
McKeag	**I:** 38<br>**P:** 36	**I:** 65.8 ± 9.4<br>**P:** 62.7 ± 9.0	**I:** 31 (82)<br>**P:** 29 (81)	**I:** 29.5 ± 2.4<br>**P:** 29.9 ± 5.9	NR	**I:** 6 (16)<br>**P:**10 (28)	**I:** 99.6 ± 23.8<br>**P:**35.4 ± 22.0	**I:**4.6 ± 5.4<br>**P:**4.3 ± 3.8	**I:** 4.1 ± 1.4<br>**P:** 4.6 ± 1.6	**I:**4.2 ± 3.8<br>**P:**5.0 ± 6.3	**I:** 1.1 ± 0.5<br>**P:**1.5 ± 1.5	
Schleithoff	**I:** 42<br>**P:** 51	**I:** 57 (53, 63)<br>**P:**54 (50, 62)	**I:** 52 (85)<br>**P:** 50 (80)	**I:** 26 (23.9,29)<br>**P:** 25.4 (24.3, 28.4)	NR	**I:** 9 (14)<br>**P:** 7(11)	NR	**I:** 2.5 ± 0.24<br>**P:** 3.38 ± 2.68	**I:**23.5 ± 14.6<br>**P:**27.8 ± 11.6		**I:**1.26 ± 1.94<br>**P:**1.24 ± 1.52	
Schroten	**I:** 51<br>**P:** 50	**I:** 63.5 ± 11.1<br>**P:** 64.0 ± 9.0	**I:** 46 (90)<br>**P:** 48 (96)	NR	**I:** 62 (34,102)<br>**P:**61 (29,133)	NR	**I:** 80 (75, 87)<br>**P:** 44 (39, 49)					**I:** 134 (114-159)<br>**P:** 119 (105-136)
Shedeed	**I:** 42<br>**P:** 38	**I:** 10.3 ± 4.6[a]<br>**P:**11.2 ± 3.5[a]	**I:** 27 (64)<br>**P:** 22 (58)	**I:**8.6±1.6[b]<br>**P:** 8.4±1.9[b]	**I:** 5.39 ± 2.1<br>**P:** 5.11 ± 1.9	N/A	**I:** 82.1 ± 5.7<br>**P:** 36.5 ± 16.0		**I:** 0.01236 ± 0.0094<br>**P:** 0.01381 ± 0.0009	**I:**16.7±4.62<br>**P:**38.3±6.37	**I:**1.85 ± 0.36<br>**P:** 0.07±0.03	
Witham	**I:** 42<br>**P:** 42	**I:** 78.8 ± 5.6<br>**P:** 80.6 ± 5.7	**I:** 34 (64)<br>**P:** 35 (67)	**I:** 27.2 ± 5.1<br>**P:** 27.3 ± 4.5	NR	**I:** 8(15)<br>**P:** 6(12)	NR		**I:** 2.41 ± 1.31<br>**P:** 2.65 ± 2.36			
Witte	**I:** 14<br>**P:** 14	**I:** 74.2 ± 2.8<br>**P:** 75.5 ± 3.5	NR	**I:** 27.8±2.4<br>**P:** 26.4±3.5	NR	NR	NR		**I:** 5.7 ±4.5<br>**P:** 5.1 ± 2.1			

Data presented as mean ± standard deviation or median (interquartile range), unless otherwise specified.

Data not reported in published papers were obtained directly from corresponding authors.

[a]data represents months and [b]weight (g) instead of years or BMI, respectively, for study in infants.

Abbreviations: **HF**, heart failure; **BMI**, body mass index; **I**, intervention group; **P**, placebo/control group; **NR**, not reported; **N/A**, not applicable; **25(OH)D**, 25-hydroxyvitamin D; **CRP**, C-reactive protein; **IL**, interleukin; **TNF- α**, tumor necrosis factor-alpha; **FGF-23**, fibroblast growth factor-23.

Supplementary Table 5. Risk of Bias Assessment for Individual Studies

Study	Design*	Selection bias		Performance bias		Detection bias	Attrition bias		Reporting bias	Confounding		Other bias	Pooled in meta-analysis	ROB score
		Random sequence generation	Centralized /concealed allocation	Participants blinded	Investigators blinded	Outcome assessors blinded	Drop-outs reported	Intention to treat analysis	Free of selective reporting	Groups similar at baseline	Adequate statistical analysis	Funding/ COI reported		
Boxer, 2014	Parallel	NR	Yes	Yes	Yes	NR	Yes	Yes	No	Yes	No	Yes	Yes	Mod
McKeag, 2014	Parallel	Yes	Yes	Yes	Yes	Yes	Yes	Yes	Yes	No	Partial	Yes	Yes	Low
Schleithoff, 2006	Parallel	Yes	NR	Yes	Yes	NR	Yes	No	Yes	Yes	Yes	Yes	Yes	Low
Schroten, 2013	Parallel	Yes	No	No	Yes	Yes	Yes	No	No	Yes	Yes	Yes	No[a]	High
Shedeed, 2012	Parallel	No	NR	Yes	Yes	NR	N/A	Yes	Yes	Yes	Partial	No	Yes	Mod
Witham, 2010	Parallel	Yes	Yes	Yes	Yes	NR	Yes	No	Yes	Yes	Yes	Yes	Yes	Low
Witte, 2005	Parallel	No	Yes	Yes	Yes	NR	Yes	No	No	No	No	NR	Yes	High

*all trials were parallel design RCTs (ie: randomized and with a control group) unless otherwise specified; [a]Unable to obtain all or some relevant outcome data from authors; Abbreviations: **COI**, conflict of interest; **ROB**, risk of bias; **NR**, not reported; **N/A**, not-applicable; **Mod**, moderate.

Supplementary Table 6. Egger and Begg statistical tests for assessment of publication bias

Inflammatory Marker	Number of Studies	Number of Participants	Egger's test*	Begg's test*	Beggs test* (continuity corrected)
**TNF-α (ng/L)**	5	380	0.26	0.62	0.80
**CRP (mg/L)**	3	231	0.18	0.11	0.29
**IL-10 (pg/ml)**	3	247	0.21	0.60	1.00
**Il-6 (pg/ml)**	2	154	NE	NE	NE

*Reports *p*-values calculated from Egger and Begg-Mazudumar tests for assessing small effect size.
Abbreviations: **CRP**, C-reactive protein; **TNF-α**, tumor necrosis factor-alpha; **IL**, interleukin; **NE**, not estimable.

Supplementary Table 7. GRADE assessment of the effect of vitamin D supplementation on inflammatory markers meta-analyses

Marker (Number of Studies)	Vitamin D n (%)	Placebo n (%)	Risk of bias	Inconsistency (heterogeneity)	Indirectness [a]	Imprecision [b]	SMD	Quality
TNF-α (3 RCTs)	189 (49.7)	191 (50.3)	No serious risk of bias	No serious inconsistency	Moderate indirectness	No serious imprecision	TNF-α levels were -0.21 (-0.41, -0.01) SDs lower in the vitamin D group compared to placebo	⊕⊕⊕⊕ High
IL-10 (3 RCTs)	122 (49.4)	125 (50.6)	Moderate risk of bias	Serious inconsistency	Moderate indirectness	Serious imprecision	No significant effect observed	⊕⊕ Low
CRP (3 RCTs)	111 (48.1)	120 (51.9)	No serious risk of bias	Moderate heterogeneity	No serious indirectness	Serious imprecision	No significant effect observed	⊕⊕⊕ Moderate
IL-6 (2 RCTs)	80 (51.9)	74 (48.1)	Serious risk of bias	Serious inconsistency	Serious indirectness	Serious imprecision	No significant effect observed	⊕⊕ Low

[a] Determined as serious where population, outcome measure, or intervention regimens (ie: co-supplementation or bolus versus single doses).vary significantly across studies.

[b] Determined as serious where the upper or lower 95% confidence interval is >0.5. Abbreviations: GRADE, grading of recommendations, assessment, development and evaluation; **CRP**, C-reactive protein; **TNF-α**, tumor necrosis factor-alpha; **IL**, interleukin; **SMD**, standardized mean difference; **SD/s**, standard deviation/s

Supplementary Figure 1. Funnel plots for assessment of publication bias

Tumor necrosis factor-alpha:

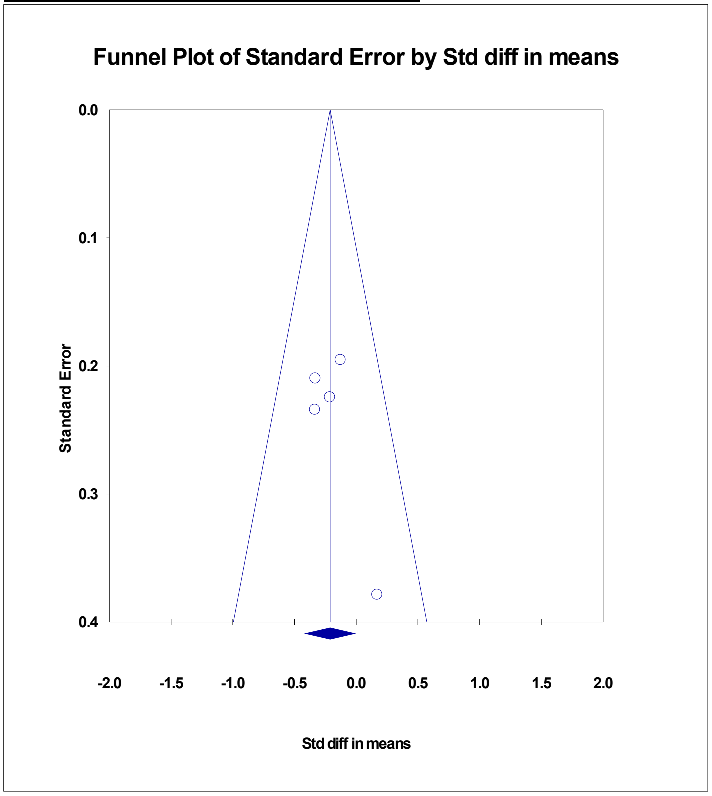

C-reactive protein:

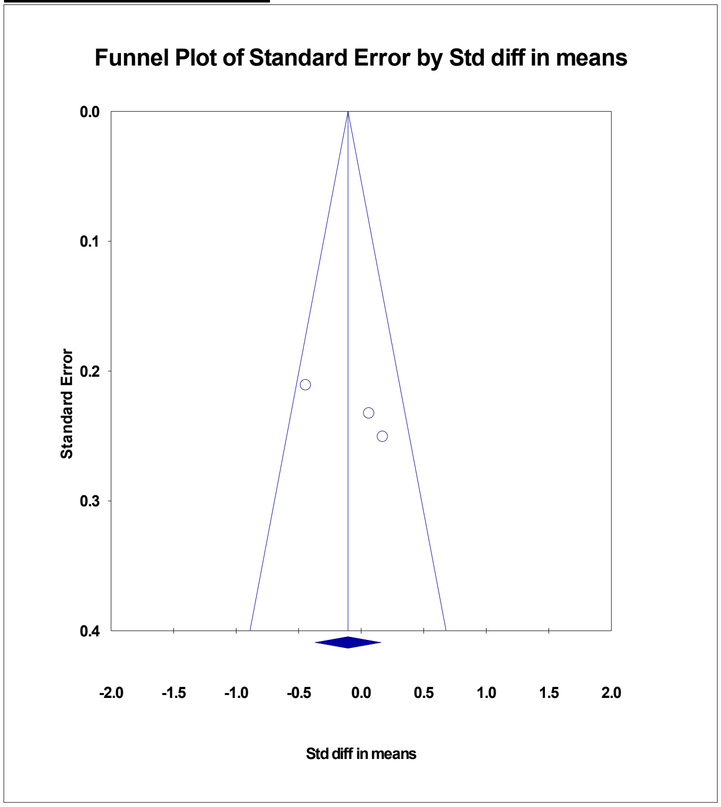

Interleukin 10:

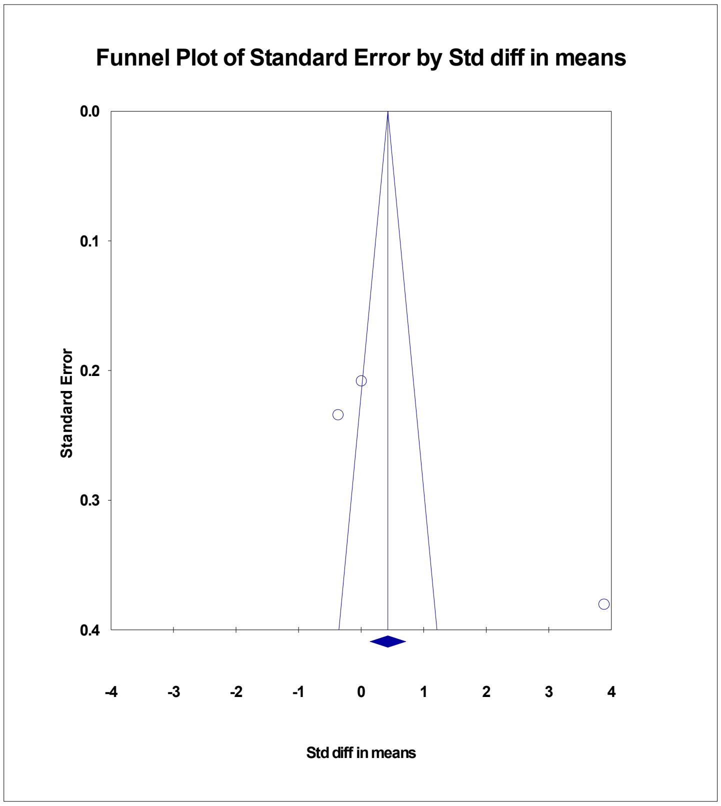

APPENDICES

Appendix 1. PRISMA 2009 Checklist

Section/topic	#	Checklist item	Reported on page #
**TITLE**			
Title	1	Identify the report as a systematic review, meta-analysis, or both.	1
**ABSTRACT**			
Structured summary	2	Provide a structured summary including, as applicable: background; objectives; data sources; study eligibility criteria, participants, and interventions; study appraisal and synthesis methods; results; limitations; conclusions and implications of key findings; systematic review registration number.	2
**INTRODUCTION**			
Rationale	3	Describe the rationale for the review in the context of what is already known.	3
Objectives	4	Provide an explicit statement of questions being addressed with reference to participants, interventions, comparisons, outcomes, and study design (PICOS).	3
**METHODS**			
Protocol and registration	5	Indicate if a review protocol exists, if and where it can be accessed (e.g., Web address), and, if available, provide registration information including registration number.	4 [CRD:42016047753]
Eligibility criteria	6	Specify study characteristics (e.g., PICOS, length of follow-up) and report characteristics (e.g., years considered, language, publication status) used as criteria for eligibility, giving rationale.	4 + Supplementary Table 1
Information sources	7	Describe all information sources (e.g., databases with dates of coverage, contact with study authors to identify additional studies) in the search and date last searched.	4
Search	8	Present full electronic search strategy for at least one database, including any limits used, such that it could be repeated.	4 (Appendix 2)
Study selection	9	State the process for selecting studies (i.e., screening, eligibility, included in systematic review, and, if applicable, included in the meta-analysis).	4
Data collection process	10	Describe method of data extraction from reports (e.g., piloted forms, independently, in duplicate) and any processes for obtaining and confirming data from investigators.	5
Data items	11	List and define all variables for which data were sought (e.g., PICOS, funding sources) and any assumptions and simplifications made.	5 + Supplementary Table 1
Risk of bias in individual studies	12	Describe methods used for assessing risk of bias of individual studies (including specification of whether this was done at the study or outcome level), and how this information is to be used in any data synthesis.	5
Summary measures	13	State the principal summary measures (e.g., risk ratio, difference in means).	5
Synthesis of results	14	Describe the methods of handling data and combining results of studies, if done, including measures of consistency (e.g., I^2) for each meta-analysis.	6

Section/topic	#	Checklist item	Reported on page #
Risk of bias across studies	15	Specify any assessment of risk of bias that may affect the cumulative evidence (e.g., publication bias, selective reporting within studies).	5
Additional analyses	16	Describe methods of additional analyses (e.g., sensitivity or subgroup analyses, meta-regression), if done, indicating which were pre-specified.	6
**RESULTS**			
Study selection	17	Give numbers of studies screened, assessed for eligibility, and included in the review, with reasons for exclusions at each stage, ideally with a flow diagram.	6 + Figure 1
Study characteristics	18	For each study, present characteristics for which data were extracted (e.g., study size, PICOS, follow-up period) and provide the citations.	6-7 + Table 1
Risk of bias within studies	19	Present data on risk of bias of each study and, if available, any outcome level assessment (see item 12).	7, 9-10
Results of individual studies	20	For all outcomes considered (benefits or harms), present, for each study: (a) simple summary data for each intervention group (b) effect estimates and confidence intervals, ideally with a forest plot.	Figures 1-3, Table 1, Supplementary Tables
Synthesis of results	21	Present results of each meta-analysis done, including confidence intervals and measures of consistency.	8-9
Risk of bias across studies	22	Present results of any assessment of risk of bias across studies (see Item 15).	7, 9-10
Additional analysis	23	Give results of additional analyses, if done (e.g., sensitivity or subgroup analyses, meta-regression [see Item 16]).	9
**DISCUSSION**			
Summary of evidence	24	Summarize the main findings including the strength of evidence for each main outcome; consider their relevance to key groups (e.g., healthcare providers, users, and policy makers).	10
Limitations	25	Discuss limitations at study and outcome level (e.g., risk of bias), and at review-level (e.g., incomplete retrieval of identified research, reporting bias).	12-13
Conclusions	26	Provide a general interpretation of the results in the context of other evidence, and implications for future research.	11, 13
**FUNDING**			
Funding	27	Describe sources of funding for the systematic review and other support (e.g., supply of data); role of funders for the systematic review.	14

From: Moher D, Liberati A, Tetzlaff J, Altman DG, The PRISMA Group (2009). Preferred Reporting Items for Systematic Reviews and Meta-Analyses: The PRISMA Statement. PLoS Med 6(6): e1000097. doi:10.1371/journal.pmed1000097

For more information, visit: **www.prisma-statement.org**.

Appendix 2. Sample OVID-MEDLINE search strategy

1. Vitamin D/	48. randomi?ed.ti,ab.
2. Vitamin D?.mp.	49. placebo.ti,ab.
3. 25OHD?.mp.	50. clinical trials as topic.sh.
4. 25 hydroxyvitamin D?.mp.	51. randomly.ti,ab.
5. 25-Hydroxyvitamin D3 1-alpha-Hydroxylase/	52. trial.ti.
6. 25-Hydroxyvitamin D? 1-alpha-Hydroxylase.mp.	53. or/46-52
7. 25 hydroxylase.mp.	54. exp animals/ not exp humans/
8. 24,25OHD?.mp.	55. 53 not 54
9. 24,25-Dihydroxyvitamin D3/	56. Meta-Analysis as Topic/
10. 24,25-Dihydroxyvitamin D?.mp.	57. meta analy$.tw.
11. exp Cholecalciferol/	58. metaanaly$.tw.
12. c?olecalciferol.mp.	59. Meta-Analysis/
13. Hydroxycholecalciferol/	60. (systematic adj (review$1 or overview$1)).tw.
14. hydroxyc?olecalciferol.mp.	61. exp Review Literature as Topic/
15. Dihydroxycholecalciferol/	62. or/56-61
16. Dihydroxyc?olecalciferol.mp.	63. cochrane.ab.
17. 1 alpha 25OHD.mp.	64. embase.ab.
18. 1a,25OHD?.mp.	65. (psychlit or psyclit).ab.
19. 1-alpha, 25 dihydroxyvitamin D?.mp.	66. (psychinfo or psycinfo).ab.
20. 1a, 25 dihydroxyvitamin D?.mp.	67. (cinahl or cinhal).ab.
21. 1-alpha hydroxylase.mp.	68. science citation index.ab.
22. 1-a, hydroxylase.mp.	69. bids.ab.
23. 1,25 hydroxyvitamin D?.mp.	70. cancerlit.ab.
24. 1,25 dihydroxyvitamin D?.mp.	71. or/63-70
25. 1,25 hydroxyc?olecalciferol.mp.	72. reference list$.ab.
26. 1,25 dihydroxyc?olecalciferol.mp.	73. bibliograph$.ab.
27. Calcitriol/	74. hand-search$.ab.
28. Calcitriol.mp.	75. relevant journals.ab.
29. Calcifediol/	76. manual search$.ab.
30. calcifediol.mp.	77. or/72-76
31. calciol.mp.	78. selection criteria.ab.
32. calcitetrol.mp.	79. data extraction.ab.
33. exp Ergocalciferol/	80. 78 or 79
34. ergocalciferol.mp.	81. Review/
35. ergosterol.mp.	82. 80 and 81
36. Dihydrotachysterol/	83. Comment/
37. dihydrotachysterol.mp.	84. Letter/
38. dihydrocalciol.mp.	85. Editorial/
39. alfacalcidol.mp.	86. animal/
40. paricalcitol.mp.	87. human/
41. vitamin D analogue.mp.	88. 86 not (86 and 87)
42. vitamin D analog.mp.	89. or/83-85,88
43. Ostelin.mp.	90. 62 or 71 or 77 or 82
44. Ostelin D?.mp.	91. 90 not 89
45. or/1-44	92. 53 or 91
46. randomi?ed controlled trial.pt.	93. 45 and 92
47. controlled clinical trial.pt.	94. limit 93 to humans

Appendix 3. Template for critical appraisal of randomised controlled trials:

**Study ID**		
**Study citation**		
**EXTERNAL VALIDITY – IS THIS STUDY AND ITS RESULTS GENERALIZABLE TO MY SYSTEMATIC REVIEW QUESTION?**		
**Patient/population/ participants**		
**N**	• Screened:<br>• Enrolled:<br>• Allocated/randomised:<br>• Assessed:<br>• Followed up:<br>• Dropped out:	
**Setting** (hospital, clinic, community, university)		
**Intervention/indicator** (type, dose, duration, intervals)		
**Comparison/control** (type, dose, duration, intervals)		
**Primary Outcome/s**		
**Secondary Outcome/s**		
**Inclusion Criteria**	Yes<br>No<br>NR	
**Exclusion Criteria**	Yes<br>No<br>NR	
**Does the study have a clearly focused question and/or PICO?**	Yes<br>Partial<br>No<br>NR	Consider if question is 'focused' in terms of:<br>- population studied<br>- intervention given/ exposure<br>- comparison(s)<br>- outcomes considered
**Does the study have specified inclusion/exclusion criteria?**	Yes<br>Partial<br>No	Consider if the inclusion or exclusion of patients was clearly defined a priori.
**If there were specified inclusion/ exclusion criteria, were these appropriate?**	Yes<br>Partial<br>No<br>N/A	Consider if the eligibility criteria used to specify the patients, interventions/ exposures and outcomes of interest.
**Were the outcomes measured appropriate?**	Yes<br>Partial<br>No<br>NR	Consider if the outcomes measured are appropriate and important outcomes.
**Was there sufficient duration of follow-up?**	Yes<br>Partial<br>No<br>NR	May need to check with clinicians sufficient durations event occurrence.

INTERNAL VALIDITY – HAS THIS STUDY BEEN CONDUCTED RIGOROUSLY IN ORDER TO REDUCE BIAS?			
SELECTION BIAS	**Did the study have an adequate method of randomisation?**	Yes<br>No<br>NR	Method of randomisation is considered adequate when patient's allocation is entirely due to chance.<br>Adequate methods include:<br>- computer-generated random numbers<br>- table of random numbers<br>- coin tossing<br>Inadequate methods include:<br>- systematic methods (DOB, case record number, day of the week presenting)<br>- sequence may be related to confounding variable<br>- allows foreknowledge of assignment. (These studies should therefore be classed as Controlled Clinical Trials rather than RCTs.)
	**Was allocation to intervention group concealed?**	Yes<br>No<br>NR	Concealment of allocation is considered adequate when the person responsible for allocation cannot influence which group a patient is randomised to.<br>Adequate methods of concealment of randomisation include:<br>- Centralised or pharmacy-controlled randomisation<br>- On-site computer based system with a randomisation sequence that is not readable until allocation<br>- Other approaches with robust methods to prevent foreknowledge of the allocation sequence to clinicians and patients<br>Inadequate approaches to concealment of randomisation<br>- Open random numbers lists<br>- Serially numbered envelopes (even sealed opaque envelopes can be subject to manipulation)
PERFORMANCE BIAS	**Were patients blind to intervention group?**	Yes<br>No<br>NR	Consider:<br>- how the study has attempted to maintain blinding<br>- if there is any indication that patients were aware of intervention group<br>- the fact that blinding is not always possible<br>- if every effort was made to achieve blinding
	**Were investigators and care providers blind to intervention group?**	Yes<br>Partial<br>No<br>NR	Consider:<br>- how the study has attempted to maintain blinding<br>- if there is any indication that investigators or care providers were aware of intervention group<br>- the fact that blinding is not always possible<br>- if every effort was made to achieve blinding

	**Aside from the experimental intervention, were the groups treated the same?**	Yes<br>Partial<br>No<br>NR	To be sure it's the intervention which is responsible for the effect.
**DETECTION BIAS**	**Were outcome assessors blind to intervention group?**	Yes<br>Partial<br>No<br>NR	Consider:<br>- If the outcome is objective (e.g. death) then blinding is less critical.<br>- If the outcome is subjective (e.g. symptoms or function) then blinding of the outcome assessor is critical.
	**Were all outcomes measured in a standard, valid and reliable way?**	Yes<br>Partial<br>No<br>NR	
	**Were outcomes assessed objectively and independently?**	Yes<br>Partial<br>No<br>NR	Independence of assessment is important where the result of one outcome may affect the interpretation of another.<br>When outcomes are objectively assessed, their independence from each other is less important.
**ATTRITION BIAS**	**What percentage of the individuals recruited into each arm of the study dropped out?**	I= %<br>C_1= %<br>C_2= %<br>C_3= %<br>NR	Consider:<br>- if all patients who entered the trial were properly accounted for and attributed at its conclusion.<br>- why patients dropped out, as well as how many.<br>- the drop out rate may be expected to be higher in studies conducted over a long period of time.
	**Were all the subjects analysed in the groups to which they were randomly allocated (ie intention to treat analysis)?**	Yes<br>No<br>NR	Consider:<br>- if analysis was as per protocol or intention to treat<br>- number of crossovers<br>- reason for crossover
**REPORT BIAS**	**Is the paper free of selective outcome reporting?**	Yes<br>Partial<br>No<br>NR	Consider:<br>- if all the planned outcomes were measured<br>- if all the measured outcomes were reported<br>- if any additional or composite outcomes were measured<br>This is difficult to determine if there isn't a protocol.
**CONFOUNDING**	**Were the groups similar at baseline with regards to key prognostic variables?**	Yes<br>Partial<br>No<br>NR	Key prognostic variable include age, sex, disease severity, inflammatory markers and vitamin D status. If the randomisation process worked, the groups should be similar, however particularly in small studies, some variations are very likely.<br>There should be some indication of whether differences between groups are clinically important. May need to check with clinician.

	**If confounding was present, was it controlled for?**	Yes<br>Partial<br>No<br>NR	Consider if any effort was made to control for confounding – Analyses were adjusted for:
**OTHER INTERNAL VALIDITY/BIAS**	**Were there any conflicts of interest in the writing or funding of this study?**	Yes<br>No<br>NR	Consider:<br>- if any of the authors are/were employed, sponsored etc by pharmaceutical companies, or have other financial/other ties<br>- if any commercial companies were involved in funding, writing, editing, data analysis or manuscript approval
	**Was the study sufficiently powered to detect any differences between the groups?**	Yes<br>Partial<br>No<br>NR	Consider:<br>- if an adequate sample size calculation was undertaken<br>- if the required sample size was recruited and retained<br>- for which outcomes the study was powered<br>- if confidence intervals include a clinically important difference, the study was underpowered<br>NB: this is less important if significant differences were found
	**For cross over studies - was the washout period adequate?**	Yes<br>No<br>NR<br>N/A	Consider the likely duration of action of the treatment being tested.
	**If statistical analysis was undertaken, was this appropriate?**	Yes<br>Partial<br>No<br>NR<br>N/A	Consider:<br>- whether the authors performed any statistical tests or just presented figures<br>- if the statistical analysis was planned a priori-<br>- if the data were analysed accordingly to the study protocol<br>- the type of data and the statistical tests used. (Please refer to the CCE workbook as required)<br>- use of parametric versus non-parametric tests; whether the data has been checked for normality<br>- if the tests used are obscure, why did the authors used them and have they included a reference<br>- if point estimates and measures of variability were presented for the primary outcome<br>- if subgroups were analysed appropriately<br>- if potential confounders were identified and taken into account in the analysis<br>- if there was any adjustment made for multiple testing<br>- if missing data was handled appropriately
**Comments**		*Add any other relevant comments, including if this is likely to influence the results of the study:*	

**What is the overall risk of bias?**	Low<br>Moderate<br>High<br>Insufficient information	*Low* **-** *All of the criteria have been fulfilled or where criteria have not been fulfilled it is very unlikely the conclusions of the study would be affected.*<br>*Moderate - Some of the criteria have been fulfilled and those criteria that have not been fulfilled may affect the conclusions of the study.*<br>*High - Few or no criteria fulfilled or the conclusions of the study are likely or very likely to be affected.*<br>*Insufficient information – not enough information provided on methodological quality to be able to determine risk of bias.*

Cited in full as: Monash Centre for Health Research and Implementation (MCHRI) Evidence Synthesis Program template for critical appraisal of a randomised controlled trial (2013), MCHRI – Monash University and Monash Health, Melbourne, Australia (*adapted from* Critical Appraisal Templates (2010) Centre for Clinical Effectiveness, Southern Health, Melbourne, Australia).
